# Association of Medicaid Eligibility With Surgical Readmission Among Medicare Beneficiaries

**DOI:** 10.1001/jamanetworkopen.2020.7426

**Published:** 2020-06-10

**Authors:** Benjamin A. Y. Cher, Andrew M. Ryan, Geoffrey J. Hoffman, Kyle H. Sheetz

**Affiliations:** 1 University of Michigan Medical School, Ann Arbor; 2 Center for Healthcare Outcomes and Policy, Ann Arbor, Michigan; 3 Department of Health Management and Policy, University of Michigan School of Public Health, Ann Arbor; 4 University of Michigan Institute for Healthcare Policy and Innovation, Ann Arbor; 5 Department of Systems, Population, and Leadership, University of Michigan School of Nursing, Ann Arbor; 6 Department of Surgery, University of Michigan Medical School, Ann Arbor

## Abstract

**Importance:**

The Centers for Medicare & Medicaid Services is beginning to consider adjusting for social risk factors, such as dual eligibility for Medicare and Medicaid, when evaluating hospital performance under value-based purchasing programs. It is unknown whether dual eligibility represents a unique domain of social risk or instead represents clinical risk unmeasured by variables available in traditional Medicare claims.

**Objective:**

To assess how dual eligibility for Medicare and Medicaid is associated with risk-adjusted readmission rates after surgery.

**Design, Setting, and Participants:**

A retrospective cohort study was conducted of 55 651 Medicare beneficiaries undergoing general, vascular, and gynecologic surgery at 62 hospitals in Michigan between January 1, 2014, and December 1, 2016. Representative cohorts were derived from traditional Medicare claims (n = 29 710) and the Michigan Surgical Quality Collaborative (MSQC) clinical registry (n = 25 941), which includes additional measures of clinical risk. Statistical analysis was conducted between April 10 and July 15, 2019. The association between dual eligibility and risk-adjusted 30-day readmission rates after surgery was compared between models inclusive and exclusive of additional measurements of clinical risk. The study also examined how dual eligibility is associated with hospital profiling using risk-adjusted readmission rates.

**Exposures:**

Dual eligibility for Medicare and Medicaid.

**Main Outcomes and Measures:**

Risk-adjusted all-cause 30-day readmission after surgery.

**Results:**

There were a total of 3986 dual-eligible beneficiaries in the Medicare claims cohort (2554 women; mean [SD] age, 72.9 [6.9] years) and 1608 dual-eligible beneficiaries in the MSQC cohort (990 women; mean [SD] age, 72.9 [6.8] years). In both data sets, higher proportions of dual-eligible beneficiaries were younger, female, and nonwhite than Medicare-only beneficiaries (Medicare claims cohort: female, 2554 of 3986 [64.1%] vs 12 879 of 25 724 [50.1%]; nonwhite, 1225 of 3986 [30.7%] vs 2783 of 25 724 [10.8%]; MSQC cohort: female, 990 of 1608 [61.6%] vs 12 578 of 24 333 [51.7%]; nonwhite, 416 of 1608 [25.9%] vs 2176 of 24 333 [8.9%]). In the Medicare claims cohort, dual-eligible beneficiaries were more likely to be readmitted (15.5% [95% CI, 13.7%-17.3%]) than Medicare-only beneficiaries (13.3% [95% CI, 12.7%-13.9%]; difference, 2.2 percentage points [95% CI, 0.4-3.9 percentage points]). In the MSQC cohort, after adjustment for more granular measures of clinical risk, dual eligibility was not significantly associated with readmission (difference, 0.6 percentage points [95% CI, –1.0 to 2.2 percentage points]). In both the Medicare claims and MSQC cohorts, adding dual eligibility to risk-adjustment models had little association with hospital ranking using risk-adjusted readmission rates.

**Conclusions and Relevance:**

This study suggests that dual eligibility for Medicare and Medicaid may reflect unmeasured clinical risk of readmission in claims data. Policy makers should consider incorporating more robust measures of social risk into risk-adjustment models used by value-based purchasing programs.

## Key Points

**Question:**

In risk-adjustment models of surgical readmissions, is dual eligibility for Medicare and Medicaid associated with all-cause 30-day readmission after surgery?

**Findings:**

In this cohort study of 55 651 Medicare beneficiaries, dual eligibility was associated with readmission when using standard claims-based risk adjustment, but the association was attenuated when adjusting for additional clinical risk factors measured by a statewide clinical registry. Adjusting for dual eligibility for Medicare and Medicaid had little association with hospital rankings by risk-adjusted readmission rate.

**Meaning:**

Dual-eligibility status may reflect unmeasured clinical risk instead of social risk factors associated with readmission after surgery.

## Teaser

This cohort study of Medicare beneficiaries undergoing general, vascular, and gynecologic surgery assesses how adjustment for dual eligibility for Medicare and Medicaid is associated with risk-adjusted readmission rates after surgery.

## Introduction

The Centers for Medicare & Medicaid Services is beginning to consider adjusting for social risk factors when evaluating hospital performance under value-based purchasing (VBP) programs.^1^ Prior work suggests that accounting for social risk factors may help to address disproportionate penalties received by hospitals caring for a large proportion of patients with high social risk.^2,3,4,5,6,7,8,9^ For example, after the Hospital Readmissions Reduction Program (HRRP) began to adjust for social risk by stratifying hospitals by their proportion of patients with dual eligibility for Medicare and Medicaid, hospitals with a greater share of dual-eligible patients were less likely to be penalized.^1,10,11^ Annual per-hospital penalties decreased by as much as $436 000,^1^ and 14% of hospitals in the highest dual-eligibility quintile no longer received penalties.^10^

However, research on the association between performance under VBP programs and social risk has focused primarily on patients with medical conditions.^1,2,3,4,5,6,7,8,9,10,11^ The effect of accounting for social risk factors in readmissions after surgical care is less clear. Surgical care is an important area of study because surgical populations are being incorporated into VBP programs with greater regularity^12^ and are already a common focus for quality improvement within hospitals.^13^ Social risk factors are established factors associated with surgical readmissions,^14,15,16^ but this work is limited to younger patient populations and may not be generalizable to the Medicare population in which alternative payment demonstrations often occur. There are also concerns about whether measures of social risk, such as dual eligibility, which is currently used by some VBP programs,^1,10,11^ may represent unmeasured clinical risk severity because variables traditionally available in Medicare claims (eg, comorbidity indices) are imperfectly associated with surgical outcomes.^17^ Instead of measuring true social risk, dual eligibility may be a proxy for clinical illness severity unmeasured by the variables available in traditional Medicare claims. Whether dual eligibility represents a unique domain of social risk, and thus represents the true consequences of adjusting for dual eligibility in risk-adjustment models of surgical readmission rates, is challenging to explore using claims data alone.

In this context, we investigated how dual eligibility for Medicare and Medicaid is associated with hospital profiling using risk-adjusted surgical readmission rates at Michigan hospitals. We examined the association between dual eligibility and risk-adjusted readmission in complementary data sets: (1) Medicare claims and (2) a statewide clinical registry inclusive of additional measures to more fully account for clinical illness severity. We then compared between the data sets how the inclusion of dual eligibility into risk-adjustment models is associated with hospital profiling using risk-adjusted readmission rates. This assessed the extent to which dual eligibility, a commonly used proxy for social risk, is independent from clinical risk and therefore a unique domain of risk for readmission after surgery.

## Methods

### Study Population

**Figure 1.**
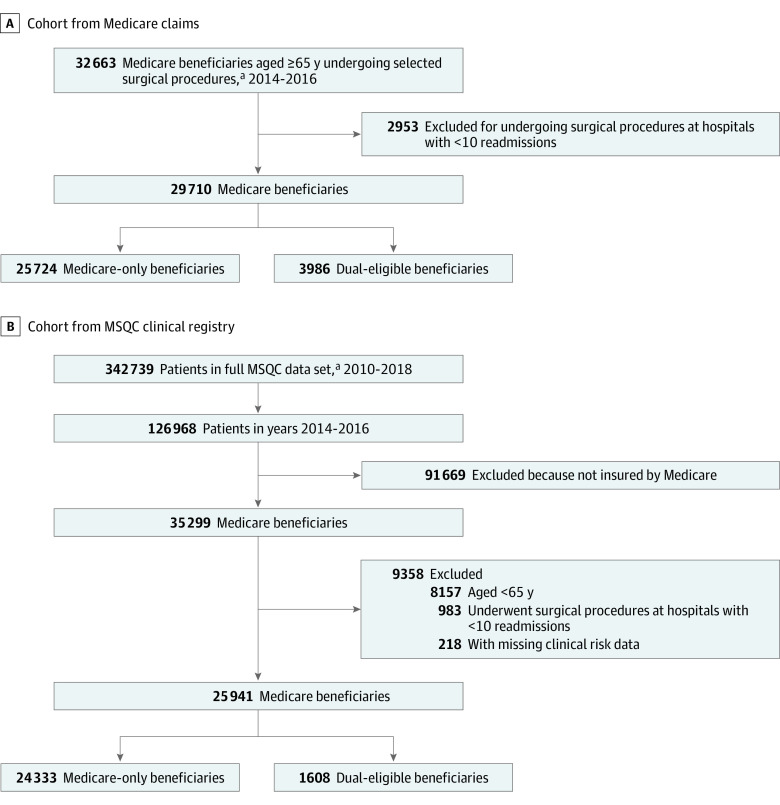
Flow Diagram Showing Sample Exclusions ^a^To build the Medicare claims cohort, we extracted data for patients undergoing the set of procedures included in the Michigan Surgical Quality Collaborative (MSQC) clinical registry, including: appendectomy, cholecystectomy, colectomy, proctectomy, esophagectomy, gastrectomy, hepatectomy, hernia repair, pancreatectomy, antireflux surgery, hysterectomy, amputations, abdominal aortic aneurysm repair, aortoiliac, carotid endarterectomy, lower extremity bypass, splenectomy, thyroidectomy, adrenalectomy, and mastectomy.

Our study included Medicare patients undergoing surgical procedures from 20 common surgical procedure categories at 62 Michigan hospitals between January 1, 2014, and December 1, 2016. Patients at hospitals with fewer than 10 readmissions during the study period were excluded from the analysis. We included the following procedure groups: appendectomy, cholecystectomy, colectomy, proctectomy, esophagectomy, gastrectomy, hepatectomy, hernia repair, pancreatectomy, antireflux surgery, hysterectomy, amputations, abdominal aortic aneurysm repair, aortoiliac, carotid endarterectomy, lower extremity bypass, splenectomy, thyroidectomy, adrenalectomy, and mastectomy. We verified each case by ensuring that *Current Procedural Terminology* codes corresponded to *International Classification of Diseases, Ninth Revision* or *International Statistical Classification of Diseases and Related Health Problems, Tenth Revision* codes as appropriate. A flow diagram of sample exclusions is presented in Figure 1. Our study was deemed exempt by the University of Michigan Institutional Review Board, which waived the requirement for patient consent because the data were deidentified. This study was designed and reported in adherence to the Strengthening the Reporting of Observational Studies in Epidemiology (STROBE) reporting guideline.

### Data Source–Medicare Claims

We extracted data from the 100% Medicare Provider Analysis and Review files for all surgical procedures of interest during the study period. We excluded patients younger than 65 years at the time of surgery. Data collected for this analysis included patient demographic characteristics, baseline comorbidities, dual eligibility, and 30-day readmission rates.

### Data Source–Michigan Surgical Quality Collaborative

We generated a similar surgical cohort using data from the Michigan Surgical Quality Collaborative (MSQC). The MSQC is a prospective clinical registry formed by a partnership between Blue Cross Blue Shield of Michigan and 73 Michigan hospitals. Data definitions and collection protocols have been described previously.^18,19^ Data were manually extracted from the electronic medical record by nurses at each hospital who were independent of financial incentives, and accuracy was ensured by rigorous training and data audits. Data collected for this analysis included patient demographic characteristics, baseline comorbidities, dual eligibility, 30-day readmission rates, and results of a preoperative clinical examination performed by the surgical team. This assessment included 2 clinical measures unavailable in traditional Medicare fee-for-service claims: functional status and American Society of Anesthesiologists (ASA) classification of physical status. Functional status measures patient independence on a 3-point ordinal scale: independent, partially dependent (requiring assistance with the instrumental activities of daily living), or totally dependent (requiring assistance with all activities of daily living). American Society of Anesthesiologists classification is widely used to describe perioperative risk and is associated with postsurgical morbidity and mortality.^20^ A higher ASA classification is associated with worse postsurgical outcomes.

### Exposure and Outcome

The study outcome was all-cause 30-day readmission after surgery. Readmission was defined as any hospitalization within 30 days of the index surgery. Exposures were dual eligibility for Medicare and Medicaid and clinical risk. Dual eligibility is commonly used as a marker of social risk.^10^ In the Medicare data set, dual eligibility was defined as eligibility for Medicaid at any point during the year in which the surgery was performed. In the MSQC data set, dual eligibility was defined as eligibility for Medicaid at the time of surgery. Clinical risk was measured using functional status and ASA classification of physical status.

### Statistical Analysis

Statistical analysis was conducted between April 10 and July 15, 2019. We determined the association between dual eligibility and risk-adjusted readmission by estimating the marginal effect of dual eligibility in the most comprehensive risk-adjustment model possible in each data set. In the Medicare data set, we modeled 30-day readmission using age, sex, case mix, dual eligibility, and Elixhauser comorbidities. We assessed the association between dual eligibility and risk-adjusted readmission by comparing the patient-level risk-adjusted readmission rate between dual-eligible beneficiaries and Medicare-only beneficiaries. We adjusted for case mix using indicator variables for each procedure group.

To address concerns about unmeasured clinical risk severity, we conducted a similar analysis in the MSQC data set, which includes a more robust set of clinical variables. We developed a model as above, with additional inclusion of functional status and ASA classification of physical status. We determined the association between dual eligibility and risk-adjusted readmission as described above.

Then, for each data set, we calculated hospital-level risk-adjusted readmission rates using models both inclusive and exclusive of dual eligibility. In each model, patients’ estimated probability of readmission was used to calculate each hospital’s expected readmission rate. For each hospital, we divided the observed number of readmissions by the expected number of readmissions. We then multiplied by the population readmission rate, inclusive of patients at all hospitals, to generate a risk-adjusted readmission rate for each hospital. We ranked hospitals by their risk-adjusted readmission rate and presented the data in a caterpillar plot with 95% CIs. Each caterpillar plot compared models inclusive and exclusive of dual eligibility, allowing us to examine how the inclusion of dual eligibility changed the distribution of the risk-adjusted readmission rate across hospitals.

We conducted sensitivity analyses to confirm the robustness of our findings. We replicated our analyses while restricting the study population to patients undergoing 2 common procedures: cholecystectomy and colectomy (to represent surgical procedures with high and low readmission risk). Then, to ensure the generalizability of our results to the entire United States, we replicated our analysis of Michigan Medicare data using a national data set. Results of sensitivity analyses are presented in eTable 1, eTable 2, and the eFigure in the Supplement.

All models used multiple logistic regression to estimate 30-day readmission rates. We used the Huber/White sandwich estimator to obtain robust variance estimates that adjust for within-hospital correlation of risk-adjusted readmission rates. We evaluated model performance using the C statistic (area under the receiver operating characteristic curve).

Statistical analyses were performed using Stata, version 15.1 (StataCorp LLC). All *P* values were from 2-sided tests, and results were deemed statistically significant at *P* < .05.

## Results

The Medicare claims cohort included 29 710 Medicare beneficiaries at 61 hospitals in Michigan, of whom 3986 were dual eligible (Table 1). Compared with Medicare-only beneficiaries, dual-eligible beneficiaries were younger (mean [SD], 72.9 [6.9] vs 75.7 [7.5] years; *P* < .001), and higher proportions of dual-eligible beneficiaries were female (2554 of 3986 [64.1%] vs 12 879 of 25 724 [50.1%]; *P* < .001), were nonwhite (1225 of 3986 [30.7%] vs 2783 of 25 724 [10.8%]; *P* < .001), and had more than 1 Elixhauser comorbidity (3671 of 3986 [92.1%] vs 21 760 of 25 724 [84.6%]; *P* < .001).

**Table 1.** Characteristics of the Study Population

Characteristic	Patients, No. (%)			
	Medicare claims cohort		MSQC clinical registry cohort	
	Medicare-only beneficiaries (n = 25 724)	Dual-eligible beneficiaries (n = 3986)^a^	Medicare-only beneficiaries (n = 24 333)	Dual-eligible beneficiaries (n = 1608)^a^
No. of patients per hospital, mean	422	65	392	26
Age, mean (SD)	75.7 (7.5)	72.9 (6.9)	74.6 (7.1)	72.9 (6.8)
Female sex	12 879 (50.1)	2554 (64.1)	12 578 (51.7)	990 (61.6)
Race/ethnicity				
White	22 749 (88.4)	2687 (67.4)	21 317 (87.6)	1073 (66.7)
Nonwhite	2783 (10.8)	1225 (30.7)	2176 (8.9)	416 (25.9)
Comorbidities				
Hypertension	20 029 (77.9)	3201 (80.3)	18 287 (75.2)	1294 (80.5)
Obesity	4407 (17.1)	771 (19.3)	8439 (34.7)	599 (37.3)
Type 2 diabetes	8523 (33.1)	1706 (42.8)	6833 (28.1)	637 (39.6)
Comorbidity count, median (IQR)^b^	3 (2-10)	3 (2-10)	2 (1-7)	2 (1-8)
ASA classification^c^				
1	NA	NA	204 (0.8)	8 (0.5)
2	NA	NA	6070 (24.9)	218 (13.6)
3	NA	NA	14 348 (59.0)	999 (60.8)
4	NA	NA	3580 (14.7)	394 (24.5)
5	NA	NA	131 (0.5)	11 (0.7)
Functional status prior to surgery^d^				
Independent	NA	NA	22 227 (91.3)	1232 (76.6)
Partially dependent	NA	NA	1571 (6.5)	263 (16.4)
Totally dependent	NA	NA	535 (2.2)	113 (7.0)

Abbreviations: ASA, American Society of Anesthesiologists; IQR, interquartile range; MSQC, Michigan Surgical Quality Collaborative; NA, not applicable.

^a^ In the Medicare data set, dual eligibility is defined as Medicaid eligibility at any point during the year in which the surgery took place. In the MSQC data set, dual eligibility is defined as Medicaid eligibility at the time of surgery.

^b^ The Medicare data set includes up to 27 recorded comorbidities, and the MSQC data set includes up to 13 recorded comorbidities.

^c^ A higher ASA classification is associated with worse postsurgical outcomes.

^d^ Partially dependent: patient requires assistance with instrumental activities of daily living; totally dependent: patient requires assistance for activities of daily living.

The MSQC cohort included 25 941 Medicare beneficiaries at 62 hospitals in Michigan, of whom 1608 were dual eligible (Table 1). As in the Medicare claims cohort, dual-eligible beneficiaries were younger (mean [SD] age, 72.9 [6.8] vs 74.6 [7.1] years), and higher proportions were female (990 of 1608 [61.6%] vs 12 578 of 24 333 [51.7%]), were nonwhite (416 of 1608 [25.9%] vs 2176 of 24 333 [8.9%]), and had more than 1 Elixhauser comorbidity (1157 of 1608 [72.0%] vs 14 181 of 24 333 [58.3%]). Dual-eligible beneficiaries were more likely than Medicare-only beneficiaries to be ASA classification 4 (394 of 1608 [24.5%] vs 3580 of 24 333 [14.7%]; *P* < .001) and less likely to be ASA classification 2 (218 of 1608 [13.6%] vs 6070 of 24 333 [24.9%]; *P* < .001). Dual-eligible beneficiaries were less likely to have independent functional status than Medicare-only beneficiaries (1232 of 1608 [76.6%] vs 22 227 of 24 333 [91.3%]; *P* < .001).

The association between dual eligibility and 30-day hospital readmission is presented in Table 2. In the Medicare claims cohort, in models adjusting for age, sex, and case mix, dual eligibility was associated with a 3.2-percentage point (pp) increase (95% CI, 1.4-5.0 pp) in readmission rate. With added adjustment for clinical comorbidities, dual eligibility was associated with a 2.2-pp increase (95% CI, 0.4-3.9 pp). In the MSQC cohort in models adjusting for age, sex, and case mix, dual eligibility was associated with a 2.0-pp increase (95% CI, 0.2-3.7 pp) in the risk-adjusted readmission rate. With added adjustment only for clinical comorbidities, the association between dual eligibility and risk-adjusted readmission was 1.2 pp (95% CI, –0.5 to 3.0 pp). With added adjustment only for clinical risk assessment, the association between dual eligibility and risk-adjusted readmission was attenuated further to 0.8 pp (95% CI, –0.9 to 2.4 pp). With added adjustment for both clinical comorbidities and clinical risk assessment, the association between dual eligibility and risk-adjusted readmission was 0.6 pp (95% CI, –1.0 to 2.2 pp).

**Table 2.** Association Between Dual Eligibility and Risk-Adjusted Readmission After Surgery at Michigan Hospitals in the Medicare Claims Cohort and MSQC Clinical Registry Cohort

Data source, risk-adjustment model^a^	Risk-adjusted readmission rate (95% CI)		
	Medicare-only beneficiaries, %	Dual-eligible beneficiaries, %	Difference, percentage points^b^
Medicare claims			
Age, sex, and case mix	13.1 (12.6 to 13.7)	16.4 (14.4 to 18.3)	3.2 (1.4 to 5.0)
Age, sex, case mix, and clinical comorbidities	13.3 (12.7 to 13.9)	15.5 (13.7 to 17.3)	2.2 (0.4 to 3.9)
MSQC clinical registry			
Age, sex, and case mix	9.3 (8.9 to 9.7)	11.3 (9.7 to 13.1)	2.0 (0.2 to 3.7)
Age, sex, case mix, and clinical comorbidities	9.3 (8.9 to 9.7)	10.5 (8.9 to 12.3)	1.2 (–0.5 to 3.0)
Age, sex, case mix, and clinical risk assessment	9.4 (9.0 to 9.8)	10.1 (8.6 to 11.7)	0.8 (–0.9 to 2.4)
Age, sex, case mix, clinical comorbidities, and clinical risk assessment^c^	9.4 (9.0 to 9.8)	10.0 (8.4 to 11.5)	0.6 (–1.0 to 2.2)

Abbreviation: MSQC, Michigan Surgical Quality Collaborative.

^a^ C statistics ranged from 0.60 to 0.64 in the Medicare data set and from 0.62 to 0.66 in the MSQC data set.

^b^ Estimated marginal effect of dual eligibility on risk-adjusted readmission rate.

^c^ Clinical risk assessment includes functional status and American Society of Anesthesiologists classification of physical status.

**Figure 2.**
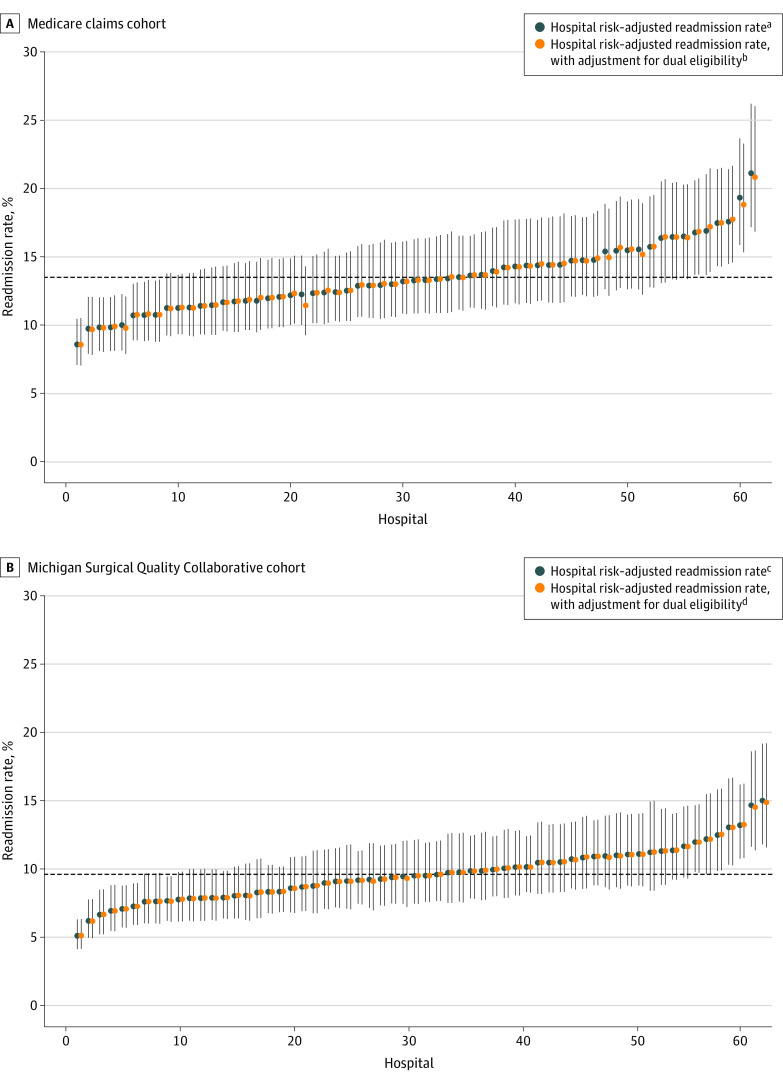
Association of Dual Eligibility With Hospital Profiling Using Risk-Adjusted Readmission Rates After Surgery at Michigan Hospitals ^a^C statistic = 6.6421. ^b^C statistic = 0.6436. ^c^C statistic = 0.6619. ^d^C statistic = 0.6620.

Adding dual eligibility to our risk-adjustment models had little association with hospital rankings in either data set (Figure 2). In Medicare claims, 27 of 61 hospitals (44.3%) did not experience a change in ranking by risk-adjusted readmission rate. Only 6 hospitals (9.8%) experienced a change greater than 1 in their ranking, with the greatest change being a decrease of 8. The average absolute change in risk-adjusted readmission rate across the 61 hospitals in our sample was 0.1 pp. The maximum decrease experienced by a single hospital was −0.8 pp, and the maximum increase experienced by a single hospital was 0.3 pp.

In the MSQC registry, 45 of 62 hospitals (72.6%) did not experience a change in rank after dual eligibility was added to the risk adjustment model. Only 1 hospital experienced a change in ranking greater than 1. The average absolute change in risk-adjusted readmission rate across the 62 hospitals in our sample was 0.03 pp. The maximum decrease experienced by a single hospital was –0.2 pp, and the maximum increase experienced by a single hospital was 0.05 pp.

Results were similar in sensitivity analyses for patients undergoing cholecystectomy or colectomy (eTable 1 in the Supplement). For patients undergoing cholecystectomy, dual eligibility was not significantly associated with 30-day hospital readmission for both cohorts. For patients undergoing colectomy, dual eligibility was associated with readmission only in the Medicare claims cohort. In both data sets, for all beneficiaries, the risk-adjusted readmission rate was higher for colectomy than for cholecystectomy.

In the national Medicare data set, we identified 836 663 surgical patients at 1940 hospitals (eTable 2 in the Supplement). In the full risk-adjustment model, dual-eligible beneficiaries were 1.8 pp (95% CI, 1.6-2.0 pp) more likely to be readmitted than Medicare-only beneficiaries. The distribution of risk-adjusted readmission rates was similar to the Michigan Medicare data set (eFigure in the Supplement). Again, there was little change in the distribution when dual eligibility was added to the risk-adjustment models. The median absolute change in ranking was 23, equivalent to a 1.2 percentile change. The largest change in ranking experienced by a single hospital was 361 (18.6 percentile difference). The mean absolute change in risk-adjusted readmission rate across the hospitals in our sample was 0.2 pp. The maximum decrease experienced by a single hospital was –1.1 pp, and the maximum increase experienced by a single hospital was 0.4 pp.

## Discussion

In this study of the association between social risk and 30-day readmission among surgical patients, we report 3 main findings. First, dual eligibility for Medicare and Medicaid was associated with a substantial increase in 30-day readmission after surgery. Second, this association was attenuated and nonsignificant when evaluated within a clinical registry with more detailed information on patient risk, suggesting that dual eligibility may be a proxy for clinical risk unmeasured by Medicare claims instead of capturing social determinants of surgical outcomes. Third, adjustment for social risk made little difference in hospitals’ relative performance on 30-day readmissions in either the Medicare claims or registry data.

Our results align with those of prior work demonstrating that social risk factors may be associated with readmission rates after surgery. For example, Joynt Maddox et al^1^ found that penalties for excess readmission after coronary artery bypass grafting for hospitals in the highest quintile of proportion of dual-eligible patients changed when the HRRP began to stratify hospitals by their proportion of dual-eligible patients. Glance et al^14^ found that the proportion of dual-eligible patients accounted for the difference in risk-adjusted surgical readmission rates between safety-net hospitals and non–safety-net hospitals. Anderson et al^15^ found that adjusting for factors such as insurance status and median income based on zip code improved the discrimination of risk-adjustment models used to estimate surgical outcomes. However, we also suggest that unmeasured clinical risk severity could explain these findings. We question whether these adjustments were successfully capturing social risk factors that varied across hospital populations or were instead adjusting for clinical risk severity that was previously unmeasured. Our results are also consistent with prior findings^14,15^ that adjustment for dual eligibility has little association with hospital ranking. We demonstrate that the prior work is generalizable to the Medicare population and a surgical cohort inclusive of several general and vascular surgery procedures.

Despite our application of robust risk-adjustment models inclusive of clinical risk, substantial variation in risk-adjusted surgical readmission rates persists across hospitals. With value-based or bundled payment models in mind, the Centers for Medicare & Medicaid Services and hospitals continue to seek explanations for this variation. For hospitals, social risk factors continue to be an important consideration when evaluating the quality of hospital care over time. The argument that social risk should be included in risk-adjustment models is valid for several reasons: patients with low socioeconomic status are more likely to be readmitted,^14,15,21^ surgical patients treated at safety-net hospitals experience higher readmission rates than patients treated at non–safety-net hospitals,^22^ and safety-net hospitals are more likely to be penalized under HRRP.^22,23^ There are also concerns about hospitals “cherry-picking” high–socioeconomic status patients to improve measured outcomes.^24^ Our findings suggest that variables available in claims data and currently available to programs such as HRRP (ie, dual eligibility) are insufficient in capturing these phenomena. Further research can explore measures of social risk^11,25^ beyond the scope of this study as possible explanations for variations in risk-adjusted surgical readmission rates. Besides dual eligibility, other measures of social risk may have more substantial associations with hospital profiling using risk-adjusted readmission rates. This work is warranted because programs such as HRRP are strongly associated with surgical readmissions—for example, HRRP began to have spillover effects on surgical conditions even before the program measured any surgical outcomes.^13^

Social risk may play a lesser, and possibly different, role in modeling surgical readmissions vs medical readmissions. Several studies of medical conditions found that risk adjustment for social variables at the patient level had substantial association with risk-adjusted readmission rates.^2,4,5,6,7,9^ In addition, one study of the HRRP policy change mentioned above that examined only medical readmissions^10^ found substantial changes in penalties associated with stratification. Further work is necessary, however, to elucidate how dual eligibility is associated with medical and surgical readmission rates differently and to understand how programs such as HRRP may be affected by inclusion of more surgical conditions.

### Limitations

Our study had some limitations. First, we did not link patient records between the Medicare and MSQC cohorts. However, these cohorts are quite similar because both data sets included the largest Michigan hospitals, and demographic and clinical variables were similar between cohorts. Second, we identified a smaller proportion of dual-eligible beneficiaries in the MSQC cohort than in the Medicare claims cohort. However, this was likely owing to the MSQC data set’s stricter definition of dual eligibility, and we are confident that the exclusion of patients was not associated with social risk. Also, demographic and clinical characteristics were similar between the dual-eligible patients identified in each data set. Third, our analysis accounts for dual eligibility at the individual level, while programs such as HRRP account for dual eligibility at the hospital level by stratifying based on hospitals’ proportions of dual-eligible patients. However, our goal was to generate results applicable to any VBP program by determining the extent to which a patient’s dual-eligibility status reflects their unmeasured clinical risk. Fourth, our analysis did not differentiate between planned and unplanned readmissions. However, planned readmissions are rare for most hospitalizations, particularly surgical procedures.^26,27^ Fifth, we identified lower readmission rates in the MSQC data set than in Medicare claims, perhaps because the MSQC data set included readmissions only to the same hospital. However, we identified similar patterns of associations between the 2 data sets, and our goal was not to directly compare readmission rates between the 2 data sets.

## Conclusions

Our results demonstrated that adjusting for dual eligibility for Medicare and Medicaid may not account for social risk factors associated with readmission after surgery. Instead of representing social risk factors associated with surgical outcomes, dual eligibility was a proxy for clinical risk severity unmeasured by variables traditionally available in Medicare claims. In addition, dual eligibility had little association with hospital ranking using risk-adjusted readmission rates. Policy makers interested in accounting for the social determinants of health in risk-adjustment models used by VBP programs should consider incorporating additional measures of social risk.

## Notes

Supplement. **eTable 1.** Association Between Dual Eligibility and Risk-Adjusted Readmission After Surgery at Michigan Hospitals, in Medicare Claims Cohort and MSQC Clinical Registry Cohort, for Cholecystectomy and Colectomy **eTable 2.** Association Between Dual Eligibility and Risk-Adjusted Readmission After Surgery, in National Medicare Claims Cohort **eFigure.** Impact of Adjusting for Dual Eligibility on Hospital Profiling Using Risk-Adjusted Readmission Rates After Surgery, in National Medicare Claims Cohort
