# Supplementary material for: Association of Medicaid Eligibility With Surgical Readmission Among Medicare Beneficiaries
Source: JAMA Netw Open. 2020 Jun 10;3(6):e207426. doi: 10.1001/jamanetworkopen.2020.7426 (PMC7287571; doi:10.1001/jamanetworkopen.2020.7426)
Supplement: Supplement. — eTable 1. Association Between Dual Eligibility and Risk-Adjusted Readmission After Surgery at Michigan Hospitals, in Medicare Claims Cohort and MSQC Clinical Registry Cohort, for Cholecystectomy and Colectomy eTable 2. Association Between Dual Eligibility and Risk-Adjusted Readmission After Surgery, in National Medicare Claims Cohort eFigure. Impact of Adjusting for Dual Eligibility on Hospital Profiling Using Risk-Adjusted Readmission Rates After Surgery, in National Medicare Claims Cohort [file jamanetwopen-3-e207426-s001.pdf]

# Supplementary Online Content

Cher BAY, Ryan AM, Hoffman GJ, Sheetz KH. Association of Medicaid eligibility with surgical readmission among Medicare beneficiaries. *JAMA Netw Open.* 2020;3(6):e207426. doi:10.1001/jamanetworkopen.2020.7426

**eTable 1.** Association Between Dual Eligibility and Risk-Adjusted Readmission After Surgery at Michigan Hospitals, in Medicare Claims Cohort and MSQC Clinical Registry Cohort, for Cholecystectomy and Colectomy
**eTable 2.** Association Between Dual Eligibility and Risk-Adjusted Readmission After Surgery, in National Medicare Claims Cohort
**eFigure.** Impact of Adjusting for Dual Eligibility on Hospital Profiling Using Risk-Adjusted Readmission Rates After Surgery, in National Medicare Claims Cohort

This supplementary material has been provided by the authors to give readers additional information about their work.

© 2020 Cher BAY et al. *JAMA Network Open.*

**eTable 1.** Association Between Dual Eligibility and Risk-Adjusted Readmission After Surgery at Michigan Hospitals, in Medicare Claims Cohort and MSQC Clinical Registry Cohort, for Cholecystectomy and Colectomy

| | | | Risk-Adjusted Readmission Rate | | |
|---|---|---|---|---|---|
| **Data Source** | **Cohort** | **Risk-Adjustment Model** | **Medicare-only Beneficiaries, % (95% CI)** | **Dual-Eligible Beneficiaries, % (95% CI)** | **Difference,* pp (95% CI)** |
| Medicare Claims | Cholecys-tectomy | Age, sex | 12.3 (11.1, 13.6) | 13.7 (9.7, 17.8) | 1.41 (-3.20, 6.02) |
| | | Age, sex, clinical comorbidities | 12.4 (11.2, 13.6) | 13.0 (9.2, 16.8) | 0.57 (-3.83, 4.97) |
| | Colectomy | Age, sex | 15.4 (13.8, 16.9) | 24.8 (19.9, 29.7) | 9.44 (4.40, 14.47) |
| | | Age, sex, clinical comorbidities | 15.7 (14.1, 17.3) | 22.4 (17.8, 27.0) | 6.72 (1.83, 11.59) |
| MSQC Clinical Registry | Cholecys-tectomy | Age, sex | 10.1 (9.4, 10.8) | 15.8 (1.0, 30.6) | 5.66 (-8.93, 20.26) |
| | | Age, sex, clinical comorbidities | 10.2 (9.6, 10.9) | 14.0 (-0.8, 28.8) | 3.80 (-11.0, 18.6) |
| | | Age, sex, clinical risk assessment | 10.3 (9.4, 11.2) | 14.4 (0.8, 28.1) | 4.10 (-9.35, 17.55) |
| | | Age, sex, clinical comorbidities, clinical risk assessment | 10.4 (9.7, 11.1) | 13.5 (-0.6, 27.6) | 3.10 (-10.99, 17.19) |
| | Colectomy | Age, sex | 13.4 (11.4, 15.5) | 17.5 (5.1, 30.0) | 4.10 (-8.61, 16.81) |
| | | Age, sex, clinical comorbidities | 13.5 (11.3, 15.7) | 16.7 (5.7, 27.7) | 3.25 (-7.94, 14.45) |
| | | Age, sex, clinical risk assessment | 13.7 (11.5, 15.8) | 16.7 (4.7, 28.7) | 3.03 (-0.95, 15.5) |
| | | Age, sex, clinical comorbidities, clinical risk assessment | 13.6 (11.4, 15.9) | 16.6 (5.4, 27.7) | 2.96 (-8.69, 14.50) |

MSQC = Michigan Surgical Quality Collaborative
CI = confidence interval; pp = percentage points
C-statistics ranged from 0.53 to 0.63 in the Medicare dataset, and from 0.58 to 0.66 in the MSQC dataset.
*Marginal effect of dual-eligibility on risk-adjusted readmission rate.

© 2020 Cher BAY et al. *JAMA Network Open.*

**eTable 2.** Association Between Dual Eligibility and Risk-Adjusted Readmission After Surgery, in National Medicare Claims Cohort

| Data Source | Cohort | Risk-adjustment model | Risk-Adjusted Readmission Rate | | |
|---|---|---|---|---|---|
| | | | Medicare-only Beneficiaries, % (95% CI) | Dual-Eligible Beneficiaries, % (95% CI) | Difference,* pp (95% CI) |
| Medicare Claims | All surgical patients | Age, sex, case mix | 12.2 (12.0, 12.3) | 15.3 (15.0, 15.5) | 3.12 (2.90, 3.36) |
| | | Age, sex, case mix, clinical comorbidities* | 12.3 (12.2, 12.5) | 14.2 (14.0, 14.4) | 1.81 (1.59, 2.03) |

MSQC = Michigan Surgical Quality Collaborative
CI = confidence interval; pp = percentage points
C-statistic = 0.6465 for model including age, sex, and case mix. C-statistic = 0.6476 with additional adjustment for clinical comorbidities.
*Marginal effect of dual-eligibility on risk-adjusted readmission rate.

© 2020 Cher BAY et al. *JAMA Network Open.*

**eFigure.** Impact of Adjusting for Dual Eligibility on Hospital Profiling Using Risk-Adjusted Readmission Rates After Surgery, in National Medicare Claims Cohort

**A. Using Traditional Risk-Adjustment Model**

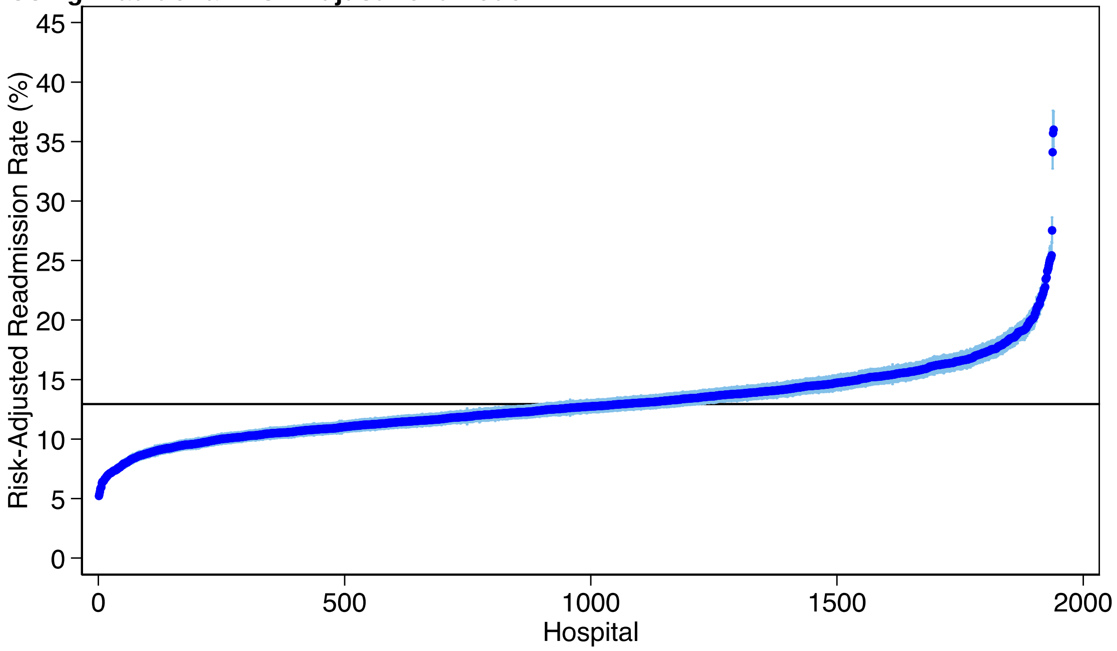


**B. Using Traditional Risk-Adjustment Model with Additional Adjustment for Dual-Eligibility**

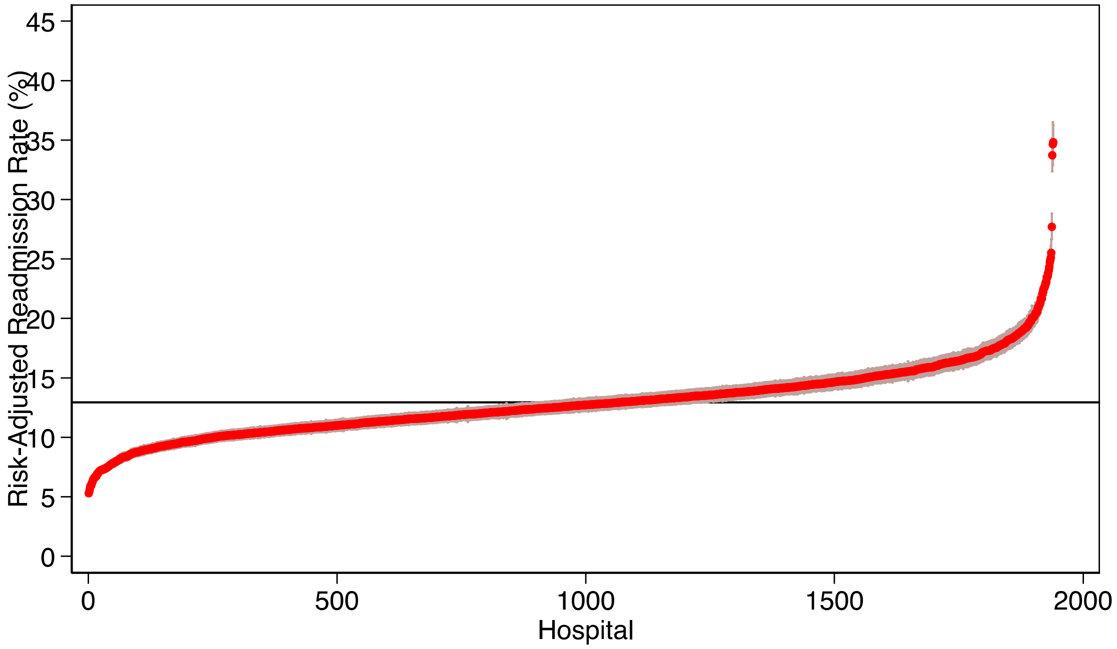


© 2020 Cher BAY et al. *JAMA Network Open.*
